# Cognitive Decline, Dementia, Alzheimer’s Disease and Presbycusis: Examination of the Possible Molecular Mechanism

**DOI:** 10.3389/fnins.2018.00394

**Published:** 2018-06-08

**Authors:** Yilin Shen, Bin Ye, Penghui Chen, Quan Wang, Cui Fan, Yilai Shu, Mingliang Xiang

**Affiliations:** ^1^Department of Otolaryngology & Head and Neck Surgery, Ruijin Hospital, Shanghai Jiao Tong University School of Medicine, Shanghai, China; ^2^Shanghai Key Laboratory of Translational Medicine on Ear and Nose Diseases, Shanghai, China; ^3^Department of Otolaryngology & Head and Neck Surgery, Shanghai Ninth People’s Hospital, Shanghai Jiao Tong University School of Medicine, Shanghai, China; ^4^Department of Otolaryngology & Head and Neck Surgery, EENT Hospital, Fudan University, Shanghai, China; ^5^Key Laboratory of Hearing Medicine, National Health and Family Planning Commission, Shanghai, China

**Keywords:** presbycusis, Alzheimer’s disease, dementia, ROS, VEGF, PGC-1α, AMPK, mitochondria

## Abstract

The incidences of presbycusis and dementia are high among geriatric diseases. Presbycusis is the general term applied to age-related hearing loss and can be caused by many risk factors, such as noise exposure, smoking, medication, hypertension, family history, and other factors. Mutation of mitochondrial DNA in hair cells, spiral ganglion cells, and stria vascularis cells of the cochlea is the basic mechanism of presbycusis. Dementia is a clinical syndrome that includes the decline of cognitive and conscious states and is caused by many neurodegenerative diseases, of which Alzheimer’s disease (AD) is the most common. The amyloid cascade hypothesis and tau hypothesis are the two major hypotheses that describe the AD pathogenic mechanism. Recent studies have shown that deposition of Aβ and hyperphosphorylation of the tau protein may cause mitochondrial dysfunction. An increasing number of papers have reported that, on one hand, the auditory system function in AD patients is damaged as their cognitive ability declines and that, on the other hand, hearing loss may be a risk factor for dementia and AD. However, the relationship between presbycusis and AD is still unknown. By reviewing the relevant literature, we found that the SIRT1-PGC1α pathway and LKB1 (or CaMKKβ)-AMPK pathway may play a role in the preservation of cerebral neuron function by taking part in the regulation of mitochondrial function. Then vascular endothelial growth factor signal pathway is activated to promote vascular angiogenesis and maintenance of the blood–brain barrier integrity. Recently, experiments have also shown that their expression levels are altered in both presbycusis and AD mouse models. Therefore, we propose that exploring the specific molecular link between presbycusis and AD may provide new ideas for their prevention and treatment.

## Introduction

The elderly population worldwide is currently approximately 900 million (de Carvalho et al., 2015). There has been a dramatic shift in the distribution of deaths from younger to older ages and from maternal, perinatal, nutritional, and communicable causes to non-communicable disease causes (Mathers and Loncar, 2005). Chronic diseases, of which dementia and presbycusis account for a large part, become more prevalent with age. This trend is exacerbated by lifestyle and behavior changes that predispose individuals to these diseases. According to the World Alzheimer Report, there were an estimated 35.6 million people with AD and other dementias in the year 2010; this number will reach 66 million by 2030 and 115 million by 2050 (Wortmann, 2012). In addition to cognitive decline, AD is also associated with secondary diseases including cardiovascular disease, tumors and sensory system dysfunctions, such as vision and hearing loss (Masters et al., 2015), which impose a heavy burden on patients as well as on society. Therefore, exploring the connections between AD and other diseases is significant for early diagnosis and prevention. As early as 1964, hearing impairment was thought to lead to mental illness caused by isolation (Kay et al., 1964). Subsequently, hearing loss was thought to be independently associated with accelerated cognitive decline and dementia (Lin et al., 2011, 2013). Hearing loss is not only the result of auditory organ damage, but may also result from central nervous system dysfunction in auditory information processing. Therefore, an increasing number of researchers have begun to explore whether improving hearing function can improve cognitive disabilities or reduce the risk of dementia later in life. Although Lin et al. (2011) found that treating hearing loss with hearing aids did not significantly decrease the risk of dementia, the risk of incident dementia did increase in participants with a hearing loss of over 25 dB. Dawes et al. (2015) suggested that hearing aids might improve cognitive performance, but this positive effect may not be the result of reducing the adverse effects of hearing loss, such as social segregation or depressed emotion. It remains unclear how presbycusis correlates with cognitive decline, dementia or AD (through social isolation caused by hearing loss, through common neuropathological pathways, or through vascular factors) and whether the relationship between them is unidirectional or bidirectional. As a result, we first review the auditory pathological changes in AD patients and AD mouse models; then, we emphasize how hearing impairment affects the incidence of cognitive decline, dementia and AD on the basis of epidemiologic evidence. Importantly, we summarize the enzymes and proteins that may contribute to the pathogenesis of both presbycusis and AD, including factors such as VEGF, SIRT1-PGC1α, and LKB1 (or CaMKKβ)-AMPK, because AD is the most common type of dementia and the molecular mechanism of AD has been extensively explored. Abnormal expression of these enzymes and proteins may cause AD (Kalaria et al., 1998; Won et al., 2010; Kumar et al., 2013) and dysfunction of cochlear hair cells (Picciotti et al., 2006; Hill et al., 2016; Xue et al., 2016). However, the specific connections between presbycusis and dementia still require further study.

## Presbycusis, Dementia and Alzheimer’s Disease

### Presbycusis

Presbycusis is the general term applied to ARHL. The risk factors for presbycusis include noise exposure, smoking, medication, hypertension, family history and other factors (Mills et al., 2009). According to statistics from the World Health Organization (data from World health Organization [WHO], 2017), a person whose hearing thresholds are over 25 dB in both ears is said to have hearing loss. Hearing loss can be classified as mild, moderate, severe, or profound. Nearly one-third of people over 65 years of age suffer from disabling hearing loss. This disorder is characterized by hearing sensitivity reduction (particularly at high frequencies), reduced understanding of speech in noisy environments, delayed central processing of acoustic information, and impaired localization of sound sources (Tavanai and Mohammadkhani, 2017). Severe hearing loss will affect an individual’s psychosocial status and cause social segregation, depression or loss of self-confidence. Although at the early stage, the peripheral auditory dysfunction may be the fundamental pathological change of ARHL, impairment of CAP function becomes increasingly important in late ARHL (Gates and Mills, 2005).

Many molecular theories have been proposed for the development of ARHL. One theory posits that accumulation of ROS in the mitochondria of inner ear hair cells, spiral ganglions and epithelial cells of the stria vascularis can cause further oxidative stress and mtDNA mutations (Yamasoba, 2009; Yamasoba et al., 2013). Markaryan et al. (2009) quantified mtDNA in human cochlear tissue samples, and the deletion rate of mtDNA (4977 bp) was found to reach 32%. However, the deletion rate of mtDNA (4834 bp) was found by Kong et al. (2006) to reach over 90% in the inner ear of rats treated with D-galactose, and this type of mutation can enhance the sensitivity of the inner ear to an aminoglycoside antibiotic.

### Dementia and Alzheimer’s Disease

Dementia is a chronic and progressive deterioration disease characterized by cognitive dysfunction and abnormal mental behavior. It has become the greatest global challenge for health and social care in the 21st century (Livingston et al., 2017). Two of more common types of dementia are AD, which is characterized as a progressive, unremitting and neurodegenerative disorder (Masters et al., 2015); and vascular dementia (VD), which is mainly caused by hypertension and arteriosclerosis. AD is the main type of dementia that has caught global attention. The population of people with AD and other dementias will reach 66 million by 2030 and 115 million by 2050 (Wortmann, 2012). Dementia is associated with age, and the incidence of dementia increase from 3.9/1000 person years (pyr) at an age of 60–64 to 104.8/1000 pyr at an age of over 90, which indicates that the incidence doubles every 6.3 years. Currently, Aβ, tau protein and ApoE are the three main elements that are thought to contribute to AD. The pathology of dementia, especially AD, which is focused on in the following section, includes: (1) loss of neurons in the temporal lobes and hippocampus; (2) NFTs; (3) SPs that consist of amyloid-β (Aβ); and (4) amyloidopathy of the cerebrovasculature (Hyman, 1998). NFTs and SPs are characteristic pathological changes. The amyloid cascade hypothesis and tau hypothesis are the two major hypotheses that describe the AD pathogenic mechanism. The average duration of illness is 8–10 years, but the clinically symptomatic phases are preceded by preclinical and prodromal stages that typically extend over two decades (Masters et al., 2015). Therefore, it is of great clinical significance to diagnose and prevent AD at an early stage.

## Pathological Changes of the Auditory System in Patients with AD

**FIGURE 1:**
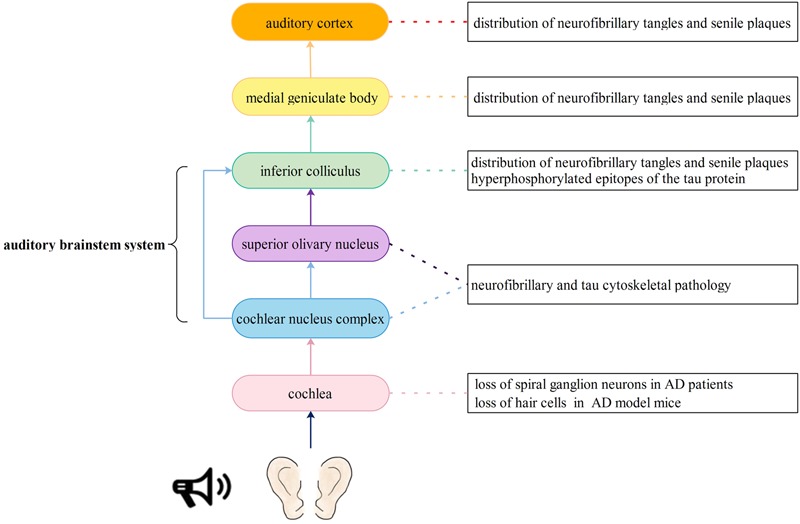
Auditory signal pathway and possible insults to the auditory nervous system caused by Alzheimer’s disease. The auditory nervous pathway originates from spiral ganglion cells in cochlea. Then, the axons of spiral ganglion cells project to the cochlear nucleus complex. Next, most of the axons from the cochlear nucleus complex cross the midline and ascend in the contralateral lateral lemniscus, terminating in the inferior colliculus and medial geniculate body. Another part of axons project to the superior olivary nucleus and then terminate in the inferior colliculus and medial geniculate body. Finally, all ascending neurons form an auditory radiation and terminate in the auditory center of the transverse temporal gyrus. Every link in the auditory pathway may display pathological changes associated with AD, thus leading to hearing impairment.

Central auditory processing dysfunction is highly evident in persons with Alzheimer’s disease (Idrizbegovic et al., 2011), and pathological changes have also been found in the auditory system, as described in the following studies. The auditory nervous pathway originates from afferent neurons called spiral ganglion cells in cochlea. Spiral ganglion cells are a type of bipolar neurons located in the Rosenthal canals of the bony modiolus. Next, the axons of spiral ganglion cells project to the cochlear nucleus complex. Then, most of the axons from the cochlear nucleus complex cross the midline and ascend in the contralateral lateral lemniscus, terminating in the inferior colliculus and medial geniculate body. Finally, all ascending neurons form an auditory radiation and terminate in the auditory center of the transverse temporal gyrus (Lonsbury-Martin et al., 2009). Neurons in different parts of the medial geniculate body are represented with the low best frequencies arranged laterally and high best frequencies arranged medially (Aitkin and Webster, 1971). Volume and quality loss of the cerebrum (meaning the sulcus is wider and deeper) as well as atrophy of the gyri have been observed in the cerebrum of AD patients using imaging methods. Consequently, we hypothesize that patients with AD might have hearing loss at both low and high frequencies if the neurons in the medial geniculate body are widely degenerated. Therefore, every link in the auditory pathway may display pathological changes associated with AD, thus leading to hearing impairment (**Figure 1**).

### Cochlear Pathology

The production and conduction of auditory signals in the cochlea mainly involve hair cells and spiral neurons. Wang and Wu (2015) reported that a significant loss of SGNs, rather than hair cells, could be found in the cochlea of 9- and 12-month-old 3xTg-AD model mice. However, O’Leary et al. (2017) found that in 5xFAD model mice (a kind of AD model mice), outer and inner hair cells also showed significantly greater losses at the apical and basal ends of the basilar membrane than wild-type mice at 15–16 months of age. Omata et al. (2016) produced new transgenic mouse models [Tg(Math1E-Aβ42Arc)1Lt/Tg(Math1E-Aβ42)1Lt], which overexpress Aβ or Aβ-related proteins in cochlear hair cells, and Tg (MathE-MAPT) 1Lt, which express human tau (2N4R) in cochlear hair cells. These transgenic mice showed auditory dysfunction, especially in high-frequency sound perception, and in these mice, expression of Aβ and tau was correlated with hearing impairment as well as hair cell loss. Consequently, it is speculated that abnormal deposition of Aβ and overexpression of tau protein in cochlea hair cells may have a synergistic effect on hearing impairment.

### Pathology of the Medial Geniculate Body

The ventral nucleus of the medial geniculate body is one of the most important relay stations in the ascending auditory pathway, and it receives fibers from neurons in the central nucleus of the inferior colliculus. Sinha et al. (1993) found that SPs and NFTs were extensively distributed throughout not only the ventral nucleus of the medial geniculate body but also the central nucleus of the inferior colliculus in AD patients at autopsy. Similarly, Rüb et al. (2016) found conspicuous AD-related cytoskeletal pathology in the inferior colliculus, superior olive and dorsal cochlear nucleus. Parvizi et al. (2001) also found β-amyloid and hyperphosphorylated epitopes of the tau protein in the inferior and superior colliculus and autonomic, monoaminergic, cholinergic, and classical reticular nucleus.

### Auditory Cortex Pathology

The human auditory cortex is mainly located in the superior temporal gyrus (Hackett, 2015). In the early stage of AD, brain atrophy involved the temporal lobe, especially the hippocampus and other brain regions, including the central auditory cortex and its related functional nuclei. In addition to the relay stations in the auditory pathway, such as the medial geniculate body and inferior colliculus, SPs and NFTs have also been observed in the primary auditory cortex and association area of the auditory cortex (Sinha et al., 1993). Recently, expression of VEGF was found to be reduced in the superior temporal, hippocampal, and brainstem regions of AD patients (Provias and Jeynes, 2014). VEGF is an important endothelial growth factor that is responsible for vascular angiogenesis, remodeling, and maintenance of the blood–brain barrier (Sondell et al., 1999). It stimulates axonal outgrowth, thus promoting cell survival and Schwann cell proliferation in the peripheral nervous system (Rosenstein et al., 2010). Moreover, VEGF repairs hair cell damage caused by noise, drugs, or certain diseases, such as otitis media and acoustic neuroma (London and Gurgel, 2014). The results mentioned above imply that reduced expression of VEGF may cause abnormalities in the structure and function of blood vessels and neurons in the auditory cortex of patients with AD, leading to a severe hearing loss. In addition to the pathological changes in auditory pathways, mouse models and patients with AD also showed increased ABR thresholds, and a greater hearing loss was related to higher adjusted relative odds of having dementia (Uhlmann et al., 1989; Goll et al., 2011; O’Leary et al., 2017).

## ARHL May Be a Risk Factor for Cognitive Decline, Dementia or AD

Defects in sensory systems, including the olfactory, visual or auditory systems, are thought to be highly associated with age-related neurodegenerative diseases (Benarroch, 2010). Impairments in peripheral and central auditory organs have been linked to accelerated cognitive decline (Bernabei et al., 2014; Amieva et al., 2015), incident cognitive impairment (Deal et al., 2017), dementia and AD (Gallacher et al., 2012; Panza et al., 2015; Taljaard et al., 2016). Therefore, audiometric testing may serve as a useful method for evaluating cognitive function, dementia and AD. After a 12-year follow-up study, Lin et al. (2011) noted that hearing loss was independently related to incident dementia after eliminating the influence of age, sex, race, education, hypertension, and other factors in 639 participants aged from 36 to 90 years old, and the attribute risk of dementia related to hearing loss reached 36.4%. In 2013, they further proposed that hearing loss was independently associated with accelerated cognitive decline (Lin et al., 2013). In addition to the peripheral auditory function, Gates et al. (2010) proposed that central auditory dysfunction and executive dysfunction might also give rise to neurodegenerative processes. CAP dysfunction in one ear was related to a sixfold increase in the risk of cognitive decline in later life after a 6-year follow-up study (Gates et al., 1996). Panza et al. (2015) concluded from many clinical trials that a CAP deficit might be an early sign of cognitive decline, and that CAP testing could be used to evaluate cognitive function in the future. Other researchers believe that hearing defects may play a part in producing mental symptoms, such as social isolation and loneliness, by reducing an individual’s contact with the outside world due to communication impairments caused by ARHL (Bennett et al., 2006). Evidence from epidemiological studies suggests that hearing loss is a modifiable factor, and with the help of appropriate and timely treatment, cognitive decline can be decelerated and daily activities can be facilitated (Behrman et al., 2014). Cognitive performance was found to be improved in auditory rehabilitation with the use of cochlear implants or hearing aids, which suggests that interventions that aim to restore hearing may be an effective way to alleviate cognitive disorders in late life (Mulrow et al., 1990; Castiglione et al., 2016). Individuals with greater hearing loss might receive the most cognitive benefit from hearing aids (Meister et al., 2015). Although many researches have shown that hearing loss may increase the risk of cognitive decline, as mentioned above, there is still much debate regarding whether there is a link between hearing loss and dementia. Gennis et al. (1991) found that hearing loss had no significant relation with cognitive function after a 5-year follow-up of 224 people aged over 60 years with no serious underlying disease. In addition, some studies have also found no evidence of improvement in behavioral symptoms, functional status, or quality of later life by providing hearing aids to hearing-impaired AD patients (Adrait et al., 2017; Nguyen et al., 2017).

In addition to the above clinical trials, patients with hearing loss have also exhibited significant reductions in the gray matter volume of the cortex related to hearing, attention and emotion, as revealed by morphometry, functional magnetic resonance (fMRI) imaging or EEG studies (Wong et al., 2010; Peelle et al., 2011; Eckert et al., 2012; Cardin, 2016). With fMRI, Peelle et al. (2011) found that a decline of peripheral auditory acuity not only led to a loss of gray matter volume in the primary auditory cortex but also contributed to down regulation of neural activity in the course of language processing. EEG studies have revealed that the greater the degree of hearing loss, the more sluggish the cortical response (Cardin, 2016). These results suggest that changes in the anatomy and function of the brain associated with hearing loss might be a reason for the increased incidence of cognitive decline and dementia. In animal experiments, Yu et al. (2011) found that when C57BL/6J mice developed profound hearing loss by 42–44 weeks of age, their cognitive function also declined, as evaluated by the Morris water maze and that the ultrastructure of the synapses of the CA3 region of the hippocampus also changed, including an increase in the synaptic cleft width and decrease of the thickness of the postsynaptic density. Therefore, we believe that there may be a certain connection between ARHL and dementia. The pathological changes of the hippocampus associated with hearing loss may lay the foundation for the incidence of cognitive decline and dementia. Similarly, Wayne and Johnsrude (2015) also proposed that there may be a common mechanism for both diseases and, to some extent, hearing loss may be an early manifestation of the underlying pathological changes.

## ARHL and AD May Result From Mitochondrial Dysfunction and Changes in Certain Signal Pathways

The role of mitochondrial dysfunction in the pathogenesis of dementia is hotly debated. Manczak et al. (2006) found that oligomeric forms of Aβ are mainly aggregated in the mitoplast (the inner membrane plus the matrix) of mitochondria inside neurons of the cortex and hippocampus. In a transgenic mouse model of AD, the hydrogen peroxide levels were greatly increased and cytochrome c oxidase activity was decreased in a transgenic mouse model of AD. These changes were directly associated with the levels of soluble Aβ, which suggest that soluble Aβ may cause more production of hydrogen peroxide and impair mitochondrial metabolism in the development and progression of AD. By contrast, aggregation of ROS may cause mtDNA mutations or deletions, and mitochondrial dysfunction is correlated with the development of ARHL (Yamasoba, 2009; Yamasoba et al., 2013). In recent decades, an increasing number of researchers have begun to pay attention to the function of the mitochondria in an effort to explore whether there is a common pathway in mitochondrial oxidative metabolism that can cause both dementia and hearing loss. If there is a common pathway between them, it may provide new ideas for preventing and treating AD as well as hearing loss.

### ROS/VEGF Pathway

Reactive oxygen species, such as O^2-^ and H_2_O_2_, can activate VEGF, which is important for angiogenesis and neuron protection. VEGF transmits signals to endothelial cells mainly through two tyrosine kinase receptors, VEGFR-1 (Flt-1) and VEGFR-2 (Flk-1) (Rosenstein et al., 2010). Research has shown that flt-1(-/-) embryos have defective sprouting from the dorsal aorta (Kearney et al., 2004). Exogenous H_2_O_2_ administered to human umbilical vein endothelial cells to simulate ROS production in cells increases mitochondrial (mtROS) through Serine 36 phosphorylation of p66Shc (Kim et al., 2017). Mitochondria in endothelial cells are also implicated in ROS signaling transactivation of VEGFR2 or VEGF-induced cell migration. Inhibition of mitochondrial respiration with rotenone and oligomycin can attenuate H_2_O_2_-induced tyrosine phosphorylation of VEGFR2 in bovine aortic endothelial cells (Chen et al., 2004). When cells are faced with a hypoxic environment, both VEGF production and VEGFR2 expression are upregulated and the generation of hydrogen peroxide is triggered by mitochondria under hypoxic conditions (Chandel et al., 1998; Colavitti et al., 2002). VEGF may also promote neurogenesis, neuronal patterning, neuroprotection and glial growth by acting on blood vessels. VEGF was found to be upregulated when the central nervous system suffered from injuries, and exogenous application of VEGF facilitated central nervous system angiogenesis (Rosenstein et al., 2010). Wang et al. (2007) found that neurogenesis and neuromigration were enhanced under ischemic condition in VEGF-overexpressing transgenic mice. In addition, the use of VEGF-specific antibodies that blocked its normal function in a stab injury model led to an increase in lesion size and reduction of angiogenic and astroglial activity in the striatum (Krum and Khaibullina, 2003).

As mentioned above, VEGF levels are reduced in the superior temporal, hippocampal, and brainstem regions in AD patients (Provias and Jeynes, 2014). VEGF is also decreased in aged ARHL mice (Picciotti et al., 2004). Therefore, as a downstream signal of mitochondrial regulation, VEGF is likely to be a common factor in the molecular mechanisms of dementia and presbycusis. Since VEGF has the function of neuroprotection, it also exerts many positive effects during the pathogenesis of AD. Expression of VEGF in the brain can increase transiently in the early stage of AD, implicating some compensatory mechanisms to counter pathological changes, such as insufficient vascularity and reduced perfusion (Kalaria et al., 1998). Exogenous VEGF was administered to transgenic mice by Burger et al. (2009) who found that VEGF modulated β-secreatase1 (BACE1) and reduced soluble Aβ1–40 and Aβ1–42. In addition, VEGF was found to bind to amyloid plaques with high affinity, most likely causing a deficiency of available and free VEGF under hypoperfusion conditions and possibly contributing to neurodegeneration and vascular dysfunction in the progression of AD (Yang et al., 2004). The decreased VEGF levels in the brain may affect the function of memory and cognition. The cognitive function of APP/PS1 mice was improved after intraperitoneal injection of VEGF (Wang et al., 2011). Intra-hippocampal administration of a VEGF receptor blocker may negatively affect long-term memory (Pati et al., 2009). Donepezi, an acetylcholinesterase inhibitor, exhibits beneficial effects in Alzheimer’s disease through activation of the PI3K/Akt/HIF-1α/VEGF pathway (Kakinuma et al., 2010). By reviewing the relevant literature, we found that VEGF is not only associated with the occurrence of dementia, but may also play an important role in many inner ear diseases. In studies, VEGF has been shown to contribute to the development of vestibular schwannoma and otitis media with effusion, and the use of a VEGF antagonist can alleviate hearing loss in these two diseases (Lim and Birck, 1971; Koutsimpelas et al., 2007; Plotkin et al., 2009; Cheeseman et al., 2011). However, the specific function of VEGF in ARHL requires further investigation. Among the many risk factors of ARHL, exposure to noise and ototoxic drugs, such as kanamycin or cisplatin, has been reported to lead to significant cochlear vascular changes, including an increase in vascular permeability, alterations in cochlear blood flow and vasoconstriction (Hawkins et al., 1972). Acoustic trauma was also found to structurally change blood vessels by disrupting the cochlear blood–barrier (Shi, 2009). In a study exploring noise-induced sensorineural damage, upregulation of VEGF was observed in the stria vascularis, spiral ligament and SGNs of guinea pigs as early as 1 day after noise exposure, and it also preceded the gradual recovery of inner ear function. Therefore, VEGF may also enhance neuron survival during the course of ARHL (Picciotti et al., 2006).

**FIGURE 2:**
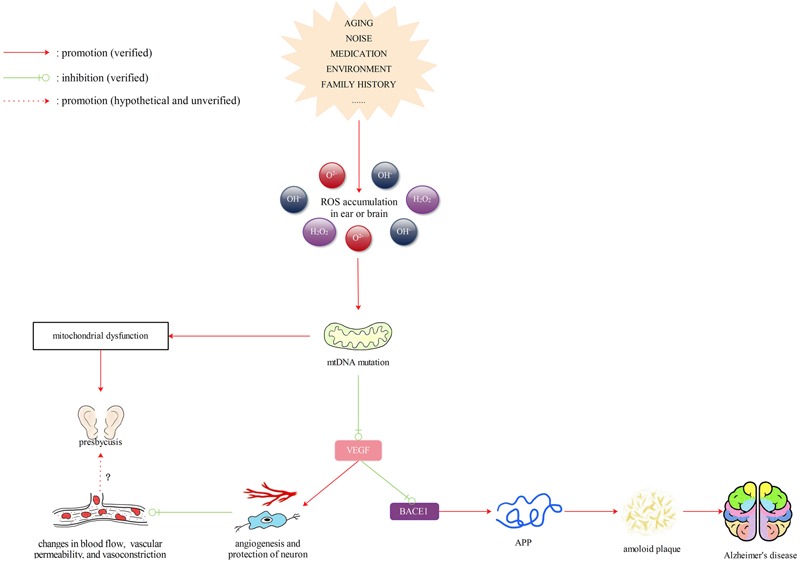
Reactive oxygen species (ROS)/VEGF pathway. Aging often causes reduced ROS clearance and increased ROS activity. Accumulation of ROS in cells can cause mtDNA mutations and mitochondrial dysfunction and then result in decreased levels of VEGF. Decreased levels of VEGF may lead to Aβ deposition and hamper the repair of hair cells and spiral ganglions in the inner ear as well as the reconstruction of blood vessels in the brain, resulting in dementia or presbycusis. Further studies are still needed to determine whether there is a time order of the occurrence of both diseases and whether hearing loss caused by a decrease of VEGF will further increase the risk of dementia and its progression.

In short, aging often causes reduced ROS clearance and increased ROS activity (Kaur et al., 2016). Accumulation of ROS in cells can cause mtDNA mutations and mitochondrial dysfunction and then result in decreased levels of VEGF. Decreased levels of VEGF may lead to Aβ deposition and hamper the repair of hair cells and spiral ganglions in the inner ear as well as the reconstruction of blood vessels in the brain, resulting in dementia or presbycusis. Further studies are needed to determine whether there is a time order of the occurrence of both diseases and whether hearing loss caused by a decrease of VEGF will further increase the risk of dementia and its progression (**Figure 2**).

### SIRT1/PGC-1α (or FNDC5) Pathway

Amyloid plaques primarily consist of Aβ1–42 and Aβ23–35 (Glenner and Wong, 1984; Gruden et al., 2007). BDNF is a versatile and multifunctional growth factor that is indispensable in a wide range of adaptive processes during human brain development, ranging from regulation of synapse formation and plasticity to neuronal differentiation and better cognitive function (Phillips et al., 1991; Park and Poo, 2013). BDNF precursors and mature BDNF were found to be reduced in the brain of patients in the pre-clinical stages of AD (Peng et al., 2005). Xia et al. (2017) found that both the protein and mRNA levels of BDNF were reduced in APP/PS1 transgenic mice as well as in the hippocampus and cerebral cortex of C57BL/6 mice after injection of Aβ1–42 oligomer. BDNF exerts protective effects in the process of neurodegeneration. In the case of AD, BDNF shifts APP processing toward the α-secretase pathway rather than the β-secretase pathway to repair the neurotoxic effects of Aβ in the brain (Holback et al., 2005). Moreover, BDNF activation is responsible for the tau dephosphorylation, thus inhibiting aggregation of the tau protein in NFTs (Murer et al., 1999; Elliott et al., 2005). A previous study showed that PPARγ coactivator-1 (PGC-1α) and its downstream activated membrane protein, FNDC5, could modulate the BDNF levels together in the PGC-1α (-/-) mouse model (Wrann et al., 2013).

PGC-1α is important in the control of cellular energy metabolic pathways (Finck and Kelly, 2006). It is more highly expressed in cells that are rich in mitochondria and is one of the factors involved in mitochondria synthesis and respiratory gene regulation (Kelly and Scarpulla, 2004). Moreover, PGC-1α also plays an important role in inhibiting neurodegeneration. PGC-1α was reported to protect against the neurodegenerative effects induced by MTPT (1-methyl-4-phenyl-1, 2, 3, 6-tetrahydropyridine) and enhance ROS detoxification (St-Pierre et al., 2006). In addition, PGC-1α can promote spinogenesis and synaptogenesis in cultured hippocampal neurons (Cheng et al., 2012). SIRT1 is a NAD+-dependent protein deacetylase located upstream of PGC-1α. It plays a crucial role in maintaining cellular homeostasis by regulating neuron survival and death, glucose metabolism, insulin sensitivity, and mitochondrial synthesis (Lagouge et al., 2006; Guarente, 2013). PCG-1α deacetylation mediated by SIRT1is required for the activation of mitochondrial fatty acid oxidation genes (McCarty et al., 2015).

Reduced levels of PGC-1α and FNDC5 were also found in Neuro-2a cells treated with Aβ oligomers *in vitro* (Xia et al., 2017). However, when PGC-1α and FNDC5 were overexpressed, the reduction in BDNF caused by Aβ oligomers was reversed, suggesting that the inhibitory action of Aβ on BDNF levels was mediated by a PGC-1α-/FNDC5-dependent pathway. With the help of BDNF treatment, deposition of Aβ in the brain was restrained and the cognitive decline was postponed in APP/PS1transgenic mice. SIRT1 can modulate Aβ metabolism by regulating the processing of amyloid precursor protein (APP) in AD progression (Kumar et al., 2013). Wang et al. (2013) found that treatment with the SIRT1-activating agent resveratrol prevented the generation of Aβ through a SIRT1-dependent mechanism. They also showed that both *in vitro* and *in vivo* experiments, downregulation of PGC-1α or PGC-1β gene transcription using specific small interfering RNA (siRNA) resulted in augmented expression of β-secreatase1 (BACE1), a protein responsible for cleaving APP. In addition, overexpression of PGC-1α decreased BACE1 protein expression in the hippocampi of transgenic mice (Wang et al., 2013). The dysfunction in the SIRT1-PGC-1α pathway not only linked to AD, but also influenced the function of hair cells and caused hearing loss. Expression of SIRT1, SIRT3, and SIRT5 was reduced in the cochlea of 22-month-old CBA/J mice (Takumida et al., 2016). A previous study revealed that SIRT1 was the direct target gene of miR-29 (Xu et al., 2014) and that expression of miR-29b was upregulated in the ARHL mouse model (Zhang et al., 2013). Xue et al. (2016) found an age-dependent downregulation in SIRT1 and PGC-1α protein levels, as well as mitochondrial dysfunction in the cochlea of aged mice. *In vitro* experiments showed that overexpression of miR-29b in HEI-OC1 cells inhibited SIRT-1 and PGC-1α protein levels, causing mitochondrial dysfunction and hair cell apoptosis, whereas in miR-29b knockdown cells, the SIRT1 and PGC-1α protein levels, as well as their mRNA levels, were all significantly upregulated (Xue et al., 2016). Additionally, overexpression of SIRT1 markedly promoted PGC-1α expression in HEI-OC1 cells, inhibited cell apoptosis, and boosted cell proliferation. Consequently, it was hypothesized that miR-29b/SIRT1/PGC-1α signaling most likely plays a role in regulating hair cells apoptosis and in the pathogenesis of ARHL.

**FIGURE 3:**
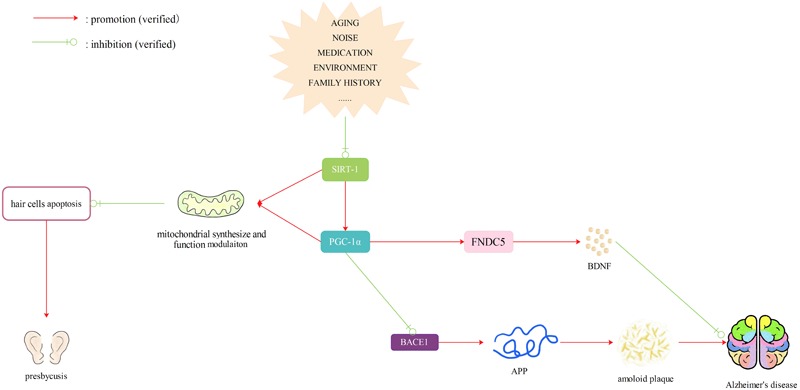
SIRTl/PGC-1α (FNDC5) pathway. Reduced expression of SIRT1- PGC-1α caused by many factors may impair the synthesis and respiratory function of mitochondria, thereby causing neuronal degeneration and cell apoptosis, thus leading to presbycusis. At the same time, reduced expression of SIRT1- PGC-lα in the brain can cause increased production of Aβ and reduced production of BDNF and thus contribute to the incidence of dementia. Therefore, SIRT1/PGC-lα (FNDC5) may be a possible drug target to prevent patients with ARHL from developing AD.

From the above research, we propose that expression of miR-29b may be increased in the inner ear with aging, which reduces the expression of its target SIRT1 and affects downstream PGC-1α and FNDC5. Reduced expression of SIRT1- PGC-1α may impair the synthesis and respiratory function of mitochondria, thereby causing neuronal degeneration and cell apoptosis, thus leading to presbycusis. At the same time, reduced expression of SIRT1- PGC-1α in the brain can cause increased production of Aβ and reduced production of BDNF and thus contribute to the incidence of AD. Though no previous study has explored the changes of miR-29b in the brains of AD patients, we believe that SIRT1/PGC-1α (FNDC5) may be a possible drug target to prevent patients with ARHL from developing AD. However, SIRT1 has recently been reported to play a completely adversarial role in the early onset of ARHL in C57BL/6 mice (Han et al., 2016). Whether SIRT1 has a positive or negative effect on ARHL requires further investigation (**Figure 3**).

### LKB1 and CaMKKβ/AMPK Pathway

AMP-activated protein kinase (AMPK) is a major regulator of cellular energy homeostasis and a central player in glucose and lipid metabolism (Daval et al., 2006; Hardie, 2008). First, when the human body is faced with hypoxia, oxidative stress or ischemia, AMPK is activated and phosphorylated by LKB1 complex or CaMKKβ, accompanied by microenvironment changes, such as an elevated AMP/ATP ratio and increased Ca^2+^ levels, respectively, thus promoting cellular glucose uptake, fatty acid β-oxidation, glucose transporter 4 synthesis and mitochondrial synthesis (Viollet et al., 2009). Activation of AMPK can lead to more fatty acids entering the mitochondria as well as stimulate the synthesis of ATP (Saha and Ruderman, 2003). It can also promote ATP synthesis by suppressing upregulation of ATPase inhibitory factor 1 (IF1) and stimulating the activity of the oxidative respiratory chain (Vazquez-Martin et al., 2013). Second, AMPK can phosphorylate the PGC-1α protein at threonine-177 and serine-538 to directly regulate mitochondrial synthesis (Jager et al., 2007) or deacetylate PGC-1α via SIRT1activation to modulate mitochondria and lipid utilization genes (Canto et al., 2010). Third, AMPK can improve mitochondrial autophagy due to reactive oxygen damage (Pauly et al., 2012). Therefore, the LKB1 (or CaMKKβ)/AMPK pathway and its association with the SIRT1/PGC-1α pathway are closely related to the function and synthesis of mitochondria.

AMP-activated protein kinase has also been shown to play a key role in regulating neurodegenerative diseases via autophagy of mitochondria (Vingtdeux et al., 2011). Transient glutamate exposure during ischemia or stroke leads to rapid and transient AMPK activation with an increase in Glucose Transporter 3 trafficking, which exerts a neuroprotective effect by increasing the ATP/AMP ratio and decreasing cytosolic Ca^2+^ levels (Weisova et al., 2011). In the progression of AD, AMPK activation may downregulate the generation of Aβ by modulating APP processing (Won et al., 2010). At the same time, AMPK is also a physiological tau kinase, and direct stimulation by AICAR (AMPK activator) inhibits tau phosphorylation (Chakraborty et al., 2008; Greco et al., 2009a,b). Moreover, AMPK activation can suppress the mTOR signaling pathway and then enhance cell autophagy and lysosomal degradation of Aβ (Anekonda et al., 2011; Vingtdeux et al., 2011). Sodium hydrosulfide was found to alleviate cell apoptosis in the auditory cortex through the CaMKKβ/AMPK pathways, suggesting that AMPK is a crucial factor in neuron degeneration of the auditory cortex in the central nervous system (Chen et al., 2017). Therefore, AMPK may be a neoteric determinant in neurodegenerative diseases and one of the possible targets for drugs aimed at AD and ARHL. Another upstream kinase of AMPK, LKB1, contributes to maintaining the development and structural stability of cochlear hair cells and stereocilia. Men et al. (2015) found that LKB1 is required for the development and maintenance of hair cell stereociliary bundles and is expressed from the cuticular plate to the nuclei of hair cells. The ABR and DPOAEs thresholds were significantly higher in Atoh1-LKB1-/- mice than those in control mice, although there were no obvious differences between mutant mice and control mice in gross morphology (Men et al., 2015). However, whether expression of LKB1 will change during the aging process and affect the process of ARHL is still unknown.

AMP-activated protein kinase is capable of inhibiting c-Jun N-terminal protein kinase (JNK) activity in neurons (Schulz et al., 2008). However, prolonged elevation of phosphorylated (p)-AMPK can trigger chronic activation of JNK, thereby causing upregulation of the proapoptotic protein Bim (Bcl-2 interacting mediator of cell death) and subsequently leading to apoptosis in neuronal and pancreatic cells, suggesting that cell fate regulation by AMPK is complex (Kefas et al., 2004; Yun et al., 2005; Weisova et al., 2011). Hill et al. (2016) proposed that long-term phosphorylation of AMPK caused by noise exposure could inversely lead to a decreased auditory function together with hair cell and synaptic ribbon loss. After CBA/J mice were exposed to 98 dB of noise, immunolabeling for p-AMPKα showed a stronger change in IHCs and marginal elevation in OHCs, while no obvious changes were detected in pillar cells. In non-exposed control animals, p-AMPKα was weak in hair cells and stronger in pillar cells. As a result, we can speculate that activation of AMPK may exert a dual influence, which may have an opposite effect on cell death and survival.

**FIGURE 4:**
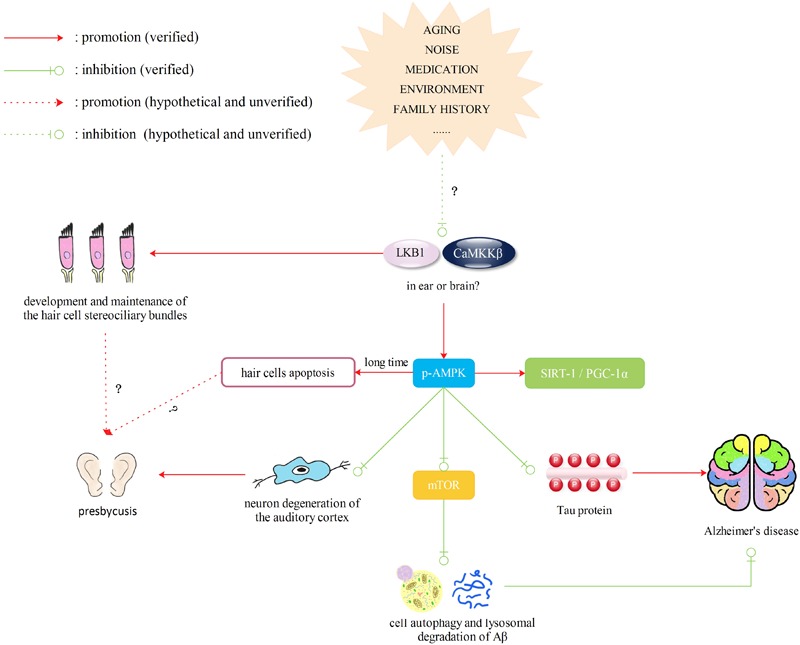
Liver kinase B1 (LKB1) and CaMKKβ/AMPK pathway. Decreased activity of AMPK will lead to abnormalities in mitochondrial synthesis and normal functions, such as oxidative respiration, as well as alterations of autophagy, which render cells unable to handle changes in the microenvironment. Decreased activity of AMPK will also cause less degradation of Aβ and more phosphorylation of tau. LKB1 and CaMKKβ are required for the activation of AMPK and are important for the stability of stereocilia in hair cells and protection of auditory cortex neurons, respectively. Although there is currently no evidence showing that LKB1 and CaMKKβ change in the ear or brain due to aging, the environment, diet or other factors, we speculate that if expression of LKB 1 and CaMKKβ is altered by the above factors, they would not only cause hearing loss but also affect downstream phosphorylation of AMPK, leading to AD. However, the activation of AMPK may exert a dual influence, which may have an opposite effect on cell death and survival.

Therefore, we can hypothesize that decreased activity of AMPK will lead to abnormalities in mitochondrial synthesis and normal functions, such as oxidative respiration, as well as alterations of autophagy, which render cells unable to handle changes in the microenvironment. Decreased activity of AMPK will also cause less degradation of Aβ and more phosphorylation of tau. LKB1 and CaMKKβ are required for the activation of AMPK and are important for the stability of stereocilia in hair cells and protection of auditory cortex neurons, respectively. Although there is currently no evidence showing that LKB1 and CaMKKβ change in the ear or brain due to aging, the environment, diet or other factors, we speculate that if expression of LKB1 and CaMKKβ is altered by the above factors, they would not only cause hearing loss but also affect downstream phosphorylation of AMPK, leading to AD. In addition, the dual effect of AMPK leads us to suggest a novel concept: AMPK may be phosphorylated in the brains of AD patients for a long time in response to intracellular environmental changes, which may damage the auditory cortex and accelerate hearing loss. Therefore, LKB1 and CaMKKβ/AMPK may also be a new target for drugs to protect patients with ARHL from dementia (**Figure 4**).

The above-mentioned publications may guide us in studying the relationship between dementia and ARHL. We speculate that expression or activity of SIRT1/PGC-1α and LKB1/AMPK may be changed by factors such as aging, the environment and medication. The expression or activity change may cause dysfunction of mitochondrial synthesis, oxidative respiration, and autophagy, leading to abnormalities of mtROS signal transduction and activation of downstream VEGFR2 transcription, followed by the development of both dementia and presbycusis. These two diseases may also influence the progression of each other as they have common pathological pathways and common targets. Undoubtedly, more research is necessary to confirm the role of these pathways. If we can find a common mechanism and verify that hearing loss is an early manifestation of dementia, it would be meaningful for the early diagnosis and prevention of dementia.

## Conclusion

Due to the non-renewable characteristic of neurons, there are no completely effective methods for curing dementia or presbycusis. With the increasing aging population, the number of people suffering from AD or other dementias and hearing disability will also increase. The factors that influence dementia and age-related hearing impairment are highly complex and involve not only age but also the environment, genetics, lifestyle, drugs and other factors. As a result, considering how to delay or prevent the incidence of dementia and how to delay the neurodegeneration in patients with dementia is important. Although pathological changes of the auditory system have been observed in patients or mice with AD, as mentioned in Section “Pathological Changes of the Auditory System in Patients with AD,” and a group of epidemiological studies have revealed that ARHL may be a risk factor for cognitive decline, dementia or AD, as mentioned in Section “ARHL May be a Risk Factor for Cognitive Decline, Dementia or AD,” the specific molecular mechanism linking these diseases is still unknown. It is also still not clear whether the relationship is unidirectional or bidirectional or if they are both the clinical manifestations of aging. Therefore, *in vivo* experiments are necessary if we are to explore the relationship between AD and ARHL at the molecular biology level. For example, we may make full use of transgenic AD mouse models, such as APP/PS1 mice, 3xTg-AD mice and 5x-FAD mice, to explore whether AD can cause more severe auditory dysfunction compared to non-transgenic mice in the same background. To explore whether ARHL can cause cognitive changes, C57BL6/J mice (a strain that develops late-onset sensorineural hearing impairment) (Noben-Trauth et al., 2003) and CBA/CaJ mice (a strain that has normal hearing throughout life) are good experiment and control groups, respectively. To our expectation, pathological changes have been found in some AD mouse models and cognitive decline has been verified in old C57BL6/J mice compared to CBA/CaJ mice of the same age, according to existing research. Although these are only patterns of manifestations, they support our belief that certain correlations really exist between ARHL and cognitive decline, dementia or AD. The next step is to explore the molecular mechanism, and with the help of a series of molecular biological techniques, we can try to validate whether the expression of proteins such as VEGF, Sirt1, PGC-1α, LKB1, CaMKKβ or AMPK has the same tendency of variation in the peripheral auditory system, central auditory system, temporal lobes and hippocampus. However, we need to overcome obstacles, such as the long time period needed for experiments, since AD and ARHL model mice require at least 6 months or more to exhibit their phenotype. In addition, we need a large sample size due to the inherent variability of the cognitive assessment made by the Morris water maze or other behavioral experiments on mice. In brief, if a common specific target or pathway is found, drugs aimed at this common target can be developed, which will be of great practical value for the early diagnosis and prevention of dementia and presbycusis.

## Author Contributions

MX and YShu: substantial contributions to the conception or design of the work. YShe, BY, PC, QW, and CF: drafting the work or revising it critically for important intellectual content agreement to be accountable for all aspects of the work in ensuring that questions related to the accuracy or integrity of any part of the work are appropriately investigated and resolved. MX: final approval of the version to be published.

## Conflict of Interest Statement

The authors declare that the research was conducted in the absence of any commercial or financial relationships that could be construed as a potential conflict of interest.

## Abbreviations

Aβ: amyloid β-protein
AD: Alzheimer’s disease
AMPK: AMP-activated protein kinase
ApoE: apolipoprotein E
ARHL: age-related hearing loss
BACE1: β-secreatase1
BDNF: brain-derived neurotrophic factor
CaMKKβ: calcium/calmodulin-dependent protein kinase kinaseβ
CAP: central auditory processing
EEG: electroencephalogram
FNDC5: fibronectin type III domain-containing protein 5
LKB1: liver kinase B1
mtDNA: mitochondrial DNA
NFTs: neurofibrillary tangles
p66Shc: 66-kilodahon isoform of Shc gene products
PCG-1α: PPARγ coactivator-1
ROS: reactive oxygen species
SGNs: spiral ganglion neurons
SPs: senile plaques
VEGF: vascular endothelial growth factor
